# “Learn and Move” Program for the Management of Chronic Musculoskeletal Pain in Primary Care: A Pilot Study

**DOI:** 10.3390/healthcare14040456

**Published:** 2026-02-11

**Authors:** Víctor Ortíz-Mallasén, Irene Llagostera-Reverter, Laura Andreu-Pejó, Marta Masiá-Ramos, Francisca Arriero-Sánchez, María Rosario Mondéjar-Martín, Cristina Cuadrado-Baca, María José Micó-López, María Jesús Valero-Chillerón, Águeda Cervera-Gasch

**Affiliations:** 1 Nursing Research Group (GIENF Code 241), Nursing Department, Universitat Jaume I, Avinguda de Vicent Sos Baynat, s/n, 12071 Castellón de La Plana, Castellón, Spain; ortizv@uji.es (V.O.-M.); pejo@uji.es (L.A.-P.); chillero@uji.es (M.J.V.-C.); cerveraa@uji.es (Á.C.-G.); 2 Integrated Health Center of Vall d’Uixó, Calle Octavi Ten, s/n, 12600 Vall d’Uixó, Castellón, Spain; masia_marram@gva.es (M.M.-R.); arriero_fra@gva.es (F.A.-S.); 3 Villena I Health Center, Calle San Francisco, 1, 03400 Villena, Alicante, Spain; mondejar_mro@gva.es (M.R.M.-M.); mico_marlop@gva.es (M.J.M.-L.); 4 Marina Española Health Center, Avenida Marina Española, s/n, 03600 Elda, Alicante, Spain; cuadrado_cri@gva.es

**Keywords:** physical therapy specialty, chronic pain, primary health care, exercise therapy, patient education as topic, neurosciences, quality of life

## Abstract

**Highlights:**

**What are the main findings?**

- A multimodal physiotherapy program that combines pain neuroscience education and therapeutic exercise is useful for the effective management of chronic musculoskeletal pain.
- The clinical benefit obtained in this program is stable, with no indications of relevant deterioration in the medium term.

**What are the implications of the main findings?**

- The results obtained suggest that this type of program is feasible for implementation in primary care and well accepted by patients. It may represent a useful alternative for the management of chronic pain in this setting.
- Given the long-term nature of chronic pain, it seems advisable to develop longer-term strategies, including maintenance activities, to consolidate and sustain the observed benefits over time.

**Abstract:**

**Background/Objectives:** Chronic musculoskeletal pain is highly prevalent in primary care and is associated with impaired quality of life and increased healthcare utilization. Multimodal physiotherapy approaches combining pain neuroscience education and therapeutic exercise are recommended; however, evidence regarding their feasibility and medium-term effects in Spanish primary care remains limited. This pilot study aimed to evaluate the usefulness of a group-based multimodal physiotherapy program on health-related quality of life and other clinical outcomes. **Methods:** A multicenter, quasi-experimental pilot study with a pre–post design and 6-month follow-up was conducted in two primary care physiotherapy units in Spain. Adults with non-specific chronic musculoskeletal pain lasting ≥6 months participated in a 12-week group-based intervention combining pain neuroscience education and therapeutic exercise. Outcomes included health-related quality of life (SF-36), pain intensity (VAS), pain catastrophizing (PCS), kinesiophobia (TSK-11), central sensitization (CSI), analgesic consumption, healthcare utilization, and patient satisfaction. Intragroup changes, effect sizes, and minimal clinically important difference were analyzed. **Results:** Thirty-seven participants completed the program, with adherence > 80% and no adverse events. Significant improvements were observed at 6 months in all outcomes, with moderate to large effect sizes (SF-36, VAS, PCS). Between 54% and 78% of participants achieved minimal clinically important differences in key domains. Improvements were maintained at follow-up without clinically relevant deterioration. **Conclusions:** This pilot study suggests that a group-based multimodal physiotherapy program is feasible and potentially effective for managing chronic musculoskeletal pain in primary care. Larger controlled trials are warranted.

## 1. Introduction

Health systems in most countries are caring for an increasing number of individuals with significant chronic diseases [1]. Among these conditions, chronic musculoskeletal pain (CMP) represents one of the most prevalent persistent disorders managed within primary care services, with prevalence estimates ranging between 25% and 30% of the general population worldwide [2,3].

CMP is classified within musculoskeletal disorders, which encompass a wide range of conditions involving the joints, muscles, bones, ligaments, tendons, and the spine. This category also includes a more heterogeneous group of disorders—such as acute and chronic conditions affecting the locomotor and connective tissue systems (e.g., systemic lupus erythematosus and spondyloarthropathies), which together reach prevalence rates of up to 48% and constitute the sixth leading cause of years lived with disability (YLDs) worldwide [4].

Health problems related to chronic pain, including CMP, represent a major public health concern due to the substantial impact they have on affected individuals, healthcare systems, and society as a whole. This burden is driven by the high levels of associated comorbidity, the considerable demand for healthcare services, the resulting disability, and the significant economic costs involved [5,6].

Available evidence indicates that individuals with CMP frequently present with comorbidities, particularly cardiovascular conditions and mental health disorders [7,8]. A recent systematic review reports that approximately 40% of individuals experiencing chronic pain exhibit clinical symptoms of anxiety and depression. However, prevalence estimates vary depending on several factors, with higher rates observed among younger populations and women, reaching levels exceeding 50% in individuals with pain conditions associated with nociplastic mechanisms, such as fibromyalgia [9].

Due to its substantial implications for both physical and emotional health, CMP adversely affects social functioning and has a clear negative impact on quality of life [10]. In this regard, previous studies have shown that CMP-related conditions, such as low back pain, represent one of the leading causes of YLDs among adult men and women worldwide [1]. Similarly, more recent evidence indicates that CMP-related conditions, such as the burden of neck pain, have increased in both incidence and YLDs over the past decade, particularly among women [11].

In addition to its health-related consequences, chronic pain represents a substantial economic burden for European healthcare systems. For example, in Norway, chronic pain is estimated to account for an annual economic burden exceeding €12 trillion, or approximately 4% of gross domestic product (GDP), with around 80% of the associated costs attributed to productivity losses [12].

One of the main challenges in addressing chronic pain, both in research and in clinical practice, lies in its multidimensional and subjective nature [13]. Accordingly, CMP appears to be influenced by a combination of physical, cognitive, emotional, and contextual factors. Emerging evidence suggests that variables such as body mass index (BMI), health-related behaviors including alcohol consumption and physical activity, and educational level also contribute to CMP [14,15,16]. Furthermore, recent studies indicate that greater knowledge of pain neurophysiology among individuals experiencing pain may be associated with lower pain intensity [16].

From this perspective, therapeutic approaches focused on pain neuroscience education (PNE) have emerged over recent decades and have demonstrated favorable outcomes. Interventions incorporating this educational component aim to provide updated information on the neurophysiology of chronic pain from a biopsychosocial perspective, thereby empowering individuals to better manage their symptoms. This process contributes to challenging maladaptive beliefs about chronic pain and facilitates its reconceptualization, promoting improved adaptation and functional adjustment in daily life [17].

Systematic reviews and meta-analyses examining PNE-based interventions for CMP have demonstrated improvements in pain intensity, disability, and pain-related knowledge, as well as reductions in pain catastrophizing and kinesiophobia, and positive changes in attitudes toward pain and physical movement [18].

However, despite these favorable outcomes and the recommendations of evidence-based clinical guidelines emphasizing multimodal approaches in which PNE is considered an essential component [17,19,20], real-world clinical practice shows that conventional physiotherapy combined with pharmacological treatment remains the most commonly used therapeutic approach in most healthcare settings [21,22].

There is growing evidence supporting the effectiveness of physiotherapy interventions based on therapeutic exercise (TE), particularly when delivered in a supervised and multicomponent manner and combined PNE, for the management of chronic musculoskeletal pain [21]. TE has demonstrated beneficial effects on pain intensity, physical function, and psychosocial factors, and the primary care setting has been identified as an appropriate and accessible environment for its implementation [22,23]. This level of care allows for resource optimization through supervised group-based interventions, which have shown promising clinical outcomes in patients with chronic pain [24,25]. However, research specifically evaluating group-based physiotherapy programs combining supervised TE and PNE in primary care settings in Spain remains limited.

Therefore, the present pilot study aims to evaluate the usefulness of a physiotherapy program based on PNE and TE, delivered in a group format within the primary care setting, in improving quality of life and other clinical outcomes.

## 2. Materials and Methods

### 2.1. Study Design

A multicenter, quasi-experimental pilot study with a pre–post design, without randomization or a control group, was conducted to determine the usefulness of a physiotherapy program based on PNE and TE.

The study was carried out between November 2023 and July 2025 and was conducted in accordance with the TREND guidelines for quasi-experimental studies [26]. This study was registered on the Open Science Framework; the registration number is DOI: 10.17605/OSF.IO/3XHKC.

### 2.2. Study Setting

The study was conducted in two physiotherapy units located in two primary care centers belonging to the Health Departments of Elda and La Plana, in the Valencian Community, Spain. The Valencian Community is organized into 24 Health Departments that provide publicly funded primary care services, including physiotherapy units within primary care centers [27]. The participating centers serve a representative sample encompassing diverse user profiles, reflecting the social reality of the Valencian Community.

### 2.3. Study Population

The study population consisted of users of the participating primary care centers who presented with nonspecific CMP of at least six months’ duration and were aged between 18 and 70 years. Individuals with pain of oncological origin, pregnant women, and those with cognitive impairments or severe psychiatric disorders were excluded. Convenience sampling was employed.

### 2.4. Outcome Measures and Instruments

The dependent variables assessed included sociodemographic variables (age, sex, weight, height, body mass index, marital status, educational level, and employment status) and clinical variables.

Clinical variables included health-related quality of life, pain intensity, pain catastrophizing, kinesiophobia, and central sensitization. Health-related quality of life was assessed using the 36-Item Short Form Health Survey (SF-36) [28]. Pain catastrophizing was evaluated with the Pain Catastrophizing Scale (PCS) [29], kinesiophobia with the 11-item Tampa Scale for Kinesiophobia (TSK-11) [30], and central sensitization with the Central Sensitization Inventory (CSI) [31]. Pain intensity was measured using a visual analogue scale (VAS) [32], and pain location, recorded using a McGill pain diagram [33]. In addition, analgesic consumption and healthcare utilization were collected using a structured self-reported questionnaire, and patient satisfaction was assessed using a standardized satisfaction questionnaire administered at the end of the intervention [34].

The psychometric properties of the instruments used are presented in Table 1.

**Figure A1:**
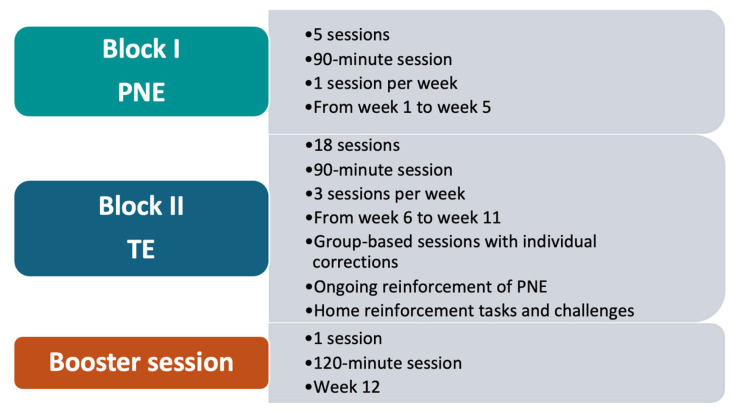
Content of the multimodal physiotherapy program. The figure illustrates the sequence of the program content, showing the two differentiated components: pain neuroscience education and therapeutic exercise. A final booster session is included to conclude the program.

The independent variable was a multimodal physiotherapy program consisting of two components (Appendix A and Figure A1): (i) PNE, delivered in five weekly sessions of 90 min each, conducted once per week between weeks 1 and 5; and (ii) TE, delivered in 18 group sessions of 90 min each, three times per week, together with reinforcement of PNE content between weeks 6 and 11. To conclude the program, a final reinforcement session lasting 120 min was conducted in week 12. The intervention was delivered by five physiotherapists across the two participating primary care centers.

Adherence to the program was calculated as the percentage of attended sessions relative to the total number of scheduled sessions. Attendance at ≥80% of the sessions was considered indicative of good adherence.

### 2.5. Data Collection

Participant recruitment was carried out by general practitioners working in primary care, who referred eligible individuals to the physiotherapy units after confirming that they met the inclusion criteria. At the physiotherapy unit, an initial assessment was conducted, during which the study was explained to participants and they were provided with the patient information sheet and written informed consent.

Data were collected at three time points: baseline assessment prior to the program (Pre), immediately after completion of the program (Post), and at 6 months following program completion (6 mo).

Questionnaires were self-administered using the REDCap platform (Research Electronic Data Capture; Vanderbilt University, Nashville, TN, USA) [35,36], through computers or tablets located in the physiotherapy units or via participants’ own mobile devices. The physiotherapists involved were present at all times to provide support and resolve any questions.

### 2.6. Statistical Analysis

Statistical analyses were performed using SPSS software (version 26; IBM Corp., Armonk, NY, USA) and JAMOVI (version 2.3.21.0; The jamovi project, Sydney, Australia). Descriptive analyses included frequencies, percentages, means, and standard deviations (SD), depending on the nature of the variables.

Given the pilot nature of the study, analyses focused on pre-intervention (Pre) to 6-month follow-up (6 mo) comparisons to evaluate medium-term effectiveness, while post-intervention (Post) assessments were used primarily to examine the stability of effects over time. To evaluate temporal changes, intragroup comparisons were conducted between baseline assessment (Pre) and the 6-month follow-up (6 mo), as well as between post-intervention assessment (Post) and the 6-month follow-up (6 mo). Paired comparisons were performed using Student’s *t*-test for paired samples. Mean differences (Δ) were calculated, and effect sizes were estimated using Cohen’s d statistic [37], interpreted according to the following thresholds: 0.15, 0.40, and 0.75 for small, medium, and large effects, respectively, together with 95% confidence intervals (95% CI). To assess the clinical relevance of the results, the percentage of patients achieving the minimal clinically important difference (MCID) for each evaluated variable was calculated [38,39,40], defined as the smallest change perceived by patients as beneficial and clinically relevant.

For dichotomous variables related to pain location, proportions were described at each of the three time points (Pre, Post, and 6 mo). Paired changes between time points were analyzed using McNemar’s test, estimating the paired odds ratio (OR) and its 95% confidence interval as measures of effect size. Absolute changes in proportions (Δ%) were also calculated as indicators of clinical impact. A significance level of *p* < 0.05 was considered statistically significant.

### 2.7. Ethical Considerations

The project was designed in accordance with Organic Law 3/2018 of 5 December on the Protection of Personal Data and Guarantee of Digital Rights [41]. No personal data allowing participant identification were collected. The research team committed to complying with the ethical principles of the Declaration of Helsinki, including beneficence, non-maleficence, autonomy, and justice.

All participants provided written informed consent prior to participation. The study received a favorable opinion from the Research Ethics Committee with Medicines of the corresponding Health Departments to which the two participating primary care centers belong (approval codes: VIFISDO V1_May 2023 and PFDCE 2024/31PI).

## 3. Results

**Figure 1:**
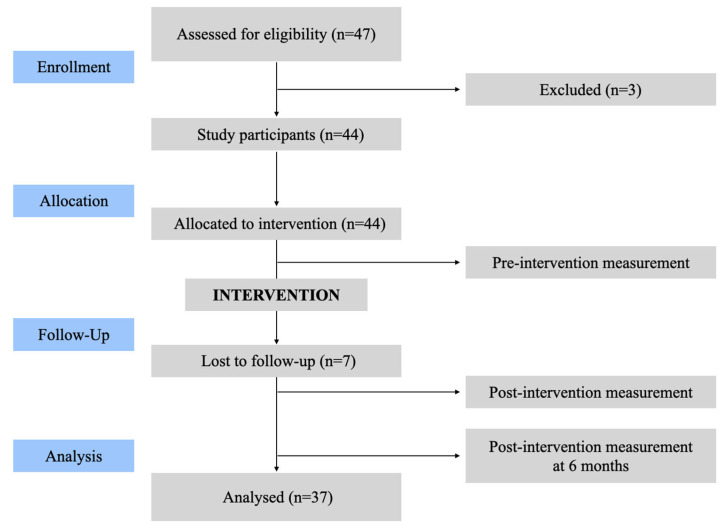
Flowchart of study participants.

Initially, 47 participants were recruited for the study, of whom 3 were excluded prior to program initiation due to not meeting the selection criteria. A total of 44 participants started the program; however, 7 participants were lost to follow-up for personal reasons. Consequently, 37 participants completed the program and were included in the final analysis (Figure 1).

Adherence to the program among participants who completed it (n = 37) exceeded 80%. In addition, no participants reported injuries or adverse events throughout the duration of the program.

### 3.1. Sociodemographic Characteristics of the Participants

The 37 patients with CMP who completed the program had a mean age of 56.19 ± 6.83 years, a mean body weight of 74.88 ± 12.07 kg, a mean height of 1.61 ± 0.05 m, and a mean BMI of 28.89 ± 4.47 kg/m^2^.

Regarding sex distribution, the sample was predominantly composed of women (94.6%; n = 35). In terms of marital status, most participants were married or in a cohabiting union (81.1%; n = 30). With respect to educational level, nearly half of the participants had completed primary education (48.6%; n = 18), and 43.2% (n = 16) were in active employment. Detailed sample characteristics are presented in Table 2.

### 3.2. Intragroup Comparisons

Table 3 presents the results obtained at each assessment time point (Pre, Post, and 6 mo), as well as the intragroup comparisons between Pre and 6 mo and between Post and 6 mo. In addition to effect size, Table 3 also shows the percentage of participants who achieved the MCID for each main variable, allowing assessment of the clinical relevance of the observed changes.

The Pre–6 mo comparison showed statistically significant improvements across all analyzed variables (Table 3). Regarding health-related quality of life (SF-36), large effect sizes were observed, particularly in the vitality (d = 0.98), role limitation physical (d = −0.90), and general health (d = −0.89) dimensions. Between 54.1% (n = 20) and 78.4% (n = 29) of participants achieved the MCID in these dimensions.

Regarding psychosocial and pain-related clinical variables, kinesiophobia (TSK-11) showed a significant reduction (Δ = 5.10; 95% CI = 2.58–7.63; d = 0.67), with 51.4% (n = 20) of participants achieving the MCID. The Central Sensitization Inventory (CSI) also demonstrated a significant improvement (Δ = 9.16; 95% CI = 4.34–13.97; d = 0.63), with 64.9% (n = 24) of participants classified as responders according to MCID criteria.

Pain catastrophizing (PCS) showed a clinically relevant reduction (Δ = 10.29; 95% CI = 7.60–12.98), with a large effect size (d = 1.27) and 64.9% (n = 24) of participants achieving the MCID. All three PCS subscales (rumination, magnification, and helplessness) exhibited large effect sizes, particularly helplessness (d = 1.15) and rumination (d = 1.04).

Pain intensity (VAS) decreased markedly (Δ = 16.13; 95% CI = 9.77–22.49), with a large effect size (d = 0.84) and 56.8% (n = 21) of participants reaching the MCID. Weekly analgesic consume also showed a notable reduction (Δ = 8.95; 95% CI = 3.80–14.11), with a moderate effect size (d = 0.57) and 73.0% (n = 27) of participants classified as responders according to MCID criteria.

On the other hand, Post–6 mo comparisons indicated an overall maintenance of the improvements achieved, with no clinically relevant deterioration. Regarding health-related quality of life (SF-36), the role emotion (Δ = 9.73; 95% CI = 4.70–14.75; d = 0.64) and general health (Δ = 5.54; 95% CI = 1.29–9.78; d = 0.43) dimensions showed the most notable changes. Overall, SF-36 dimensions remained stable, with MCID responder rates ranging from 10.8% to 35.1%. Mean differences were small and effect sizes were low across most variables, with the exception of kinesiophobia (TSK-11), which showed a moderate-to-large effect (Δ = −3.70; 95% CI = −5.25 to −2.15; d = 0.79).

The frequency of healthcare utilization showed a downward trend (Δ = −0.43; 95% CI = −0.96 to 0.10; d = −0.26). Regarding satisfaction with the intervention (CSQ-8), scores remained stable over time (Δ = −0.02; 95% CI = −0.78 to 0.72; d = −0.01); however, changes in both variables did not reach clinical relevance for the majority of participants.

**Figure 2:**
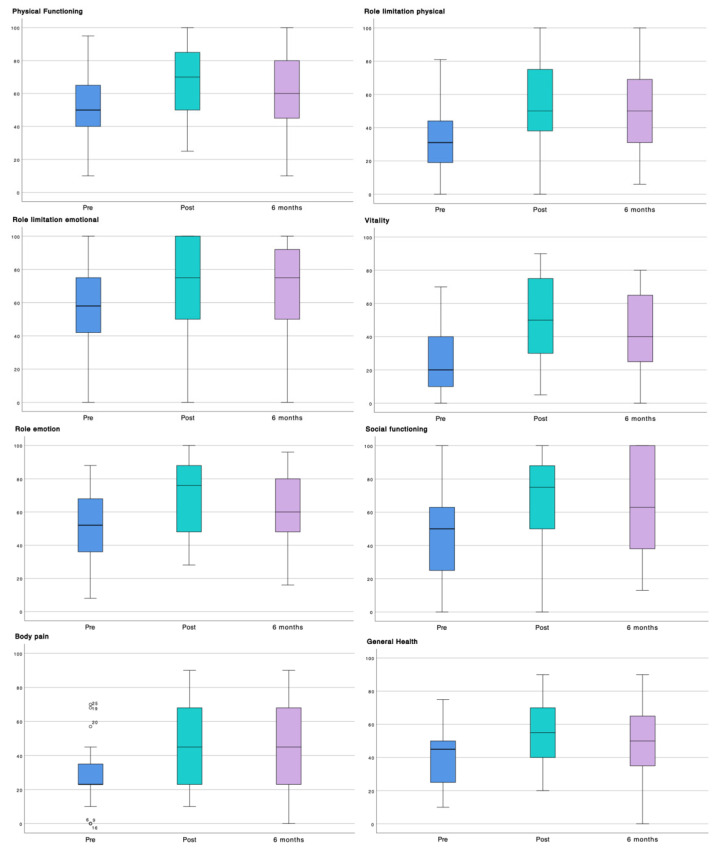
Boxplots illustrating pre–6-month clinical changes in SF-36 dimensions. The figure shows the temporal evolution of the health-related quality of life dimensions assessed using the Short Form Health Survey (SF-36), displayed through boxplots including the three assessment time points: pre-intervention (blue), post-intervention (green), and 6-month follow-up (purple). Hollow circles denote outlier values, and the numbers shown correspond to individual participants.

**Figure 3:**
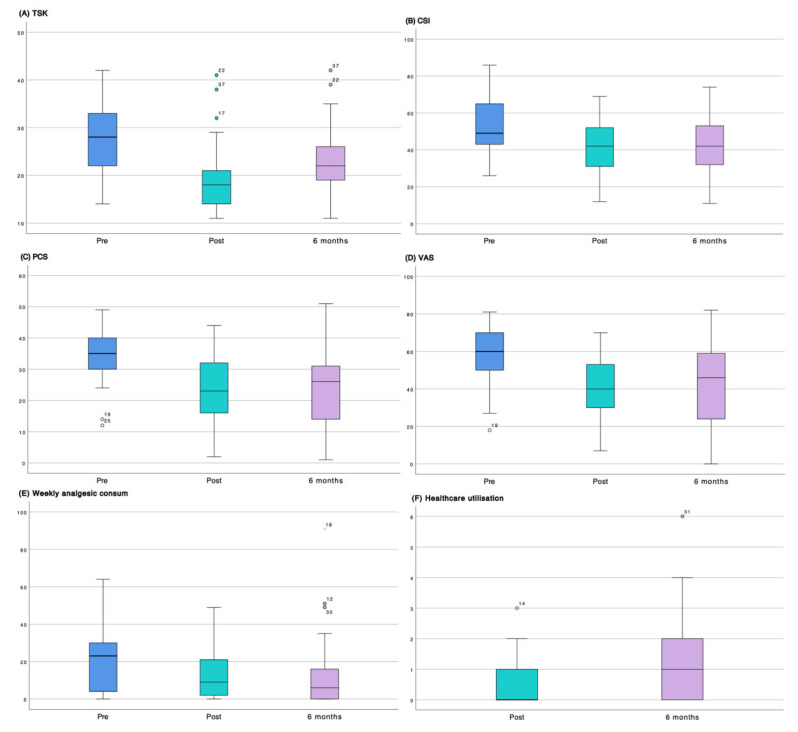
Boxplots illustrating clinical changes in the analyzed variables. The figure shows the temporal evolution of the analyzed variables using boxplots that include the three assessment time points: pre-intervention (blue), post-intervention (green), and 6-month follow-up (purple). (**A**) Boxplot of kinesiophobia (TSK-11); (**B**) boxplot of the Central Sensitization Inventory (CSI); (**C**) boxplot of pain catastrophizing (PCS); (**D**) boxplot of pain intensity (VAS); (**E**) boxplot of weekly analgesic consumption; and (**F**) boxplot of frequency of healthcare utilization. Hollow circles represent outlier values, while the asterisk (*) denotes an extreme outlier. Numbers correspond to individual participants.

The temporal evolution of each variable is summarized in the boxplots presented in Figure 2 and Figure 3, which depict the three assessment time points (Pre, Post, and 6 mo). These figures illustrate data dispersion, median values, and outliers for each of the evaluated questionnaires.

Regarding pain location, the Pre–6 mo comparison revealed a significant reduction in pain across several anatomical regions. Cervical pain decreased from 89.2% (n = 33) to 63.9% (n = 23) (Δ = −25.3%; *p* = 0.002), with an odds ratio indicating a higher likelihood of pain remission (OR = 19; 95% CI = 1.10–326.5). Low back pain showed a similar pattern (Δ = −22.6%; *p* = 0.011; OR = 9; 95% CI = 1.14–71.04).

Significant reductions were also observed in the left upper extremity (LUE) (Δ = −24.4%), as well as in the right lower extremity (RLE) and left lower extremity (LLE) (Δ = −23%), with odds ratios consistent with a decrease in pain prevalence. However, no statistically significant changes were observed in the right upper extremity (RUE), although a reduction in pain prevalence was noted (Δ = −6.8%).

During the Post–6 mo period, no statistically significant changes were identified in any of the evaluated pain locations (*p* > 0.05), although reductions in pain prevalence were observed in some regions, including the cervical (Δ = −11.8%), lumbar (Δ = −6.4%), RLE (Δ = −6.8%), and LLE (Δ = −9.5%) areas (Table 4).

Graphical representations of the percentage evolution of the different pain locations across the three assessment time points are provided in Supplementary Materials Figure S1.

## 4. Discussion

In this pilot study, we evaluated the usefulness of a multimodal physiotherapy program based on PNE and TE for improving quality of life and other related clinical outcomes in patients with CMP. The findings indicate that the program led to clinically relevant improvements across all analyzed variables, with predominantly moderate to large effect sizes and a high proportion of participants achieving the MCID during the Pre–6 mo period. Furthermore, changes observed between Post and 6 mo assessments confirmed the stability of these benefits, with no evidence of clinical deterioration and sustained improvements compared with baseline values.

### 4.1. Overall Utility of the Program

Participants reported statistically significant clinical improvements following the program across all dimensions of health-related quality of life. Changes of considerable magnitude were observed, with effect sizes ranging from moderate to large, particularly in the vitality, physical functioning, body pain, general health, and role limitation physical dimensions. In addition, more than 70% of participants achieved the MCID, further supporting the clinical relevance of the observed outcomes.

However, more moderate changes were observed in dimensions related to the psychological and social spectrum. These findings are consistent with previous evidence [42,43,44,45], which indicates that physiotherapy programs targeting patients with chronic pain and based on TE tend to produce clinically relevant improvements primarily in the physical domains of quality of life, whereas changes in mental domains are usually more modest. Our findings, in line with those reported by Galan-Martin et al. [25], confirm this pattern, although smaller effect sizes were observed in our study. Taken together, these results suggest that TE primarily influences pain-related functional limitations, reinforcing its role as a core component of physiotherapy programs for the management of CMP [42].

In line with the findings of previous studies combining TE with PNE [46,47], quality of life improved following the program. The inclusion of this educational component may have contributed to extending the impact beyond purely physical domains [42,45,46]. From this perspective, the combination of exercise and education may generate synergistic effects that translate into broader and more sustained improvements in quality of life. This suggests a transversal impact beyond pain relief alone, consistent with contemporary chronic pain models that emphasize functionality and self-efficacy [17,48].

In this context, the program was also able to improve other pain-related clinical variables, such as kinesiophobia, central sensitization, and pain catastrophizing. Moreover, more than half of the participants achieved the MCID across all three variables. These findings are consistent with clinical trials and systematic reviews demonstrating that the combination of PNE and TE leads to greater reductions in these outcomes compared with conventional treatments or exercise alone [49,50,51,52].

Regarding kinesiophobia, the instrument used (TSK-11) does not provide formal diagnostic cut-off points; however, previous studies have suggested that scores below 23 may be considered indicative of subclinical levels. Notably, following the program, participants achieved a mean score below this threshold, in line with findings from similar studies [25,53], and reflecting outcomes that are notably more favorable than those reported in several systematic reviews [18,47].

With respect to central sensitization, although a statistically significant reduction was observed following the program, levels remained elevated. This differs from findings reported in another study [25], in which participants reached values considered within the normal range and larger effect sizes were observed. In that study, the analyzed sample size was larger and participants started from considerably lower baseline scores, whereas in our sample, very high levels of central sensitization were present prior to program initiation. This greater baseline severity may have limited the magnitude of achievable change, suggesting that patients with higher levels of central sensitization may require longer interventions or additional therapeutic strategies to achieve full normalization of this variable, as suggested by the literature [54].

Pain catastrophizing was the clinical variable that showed the largest effect size following the program, which may be related to its high sensitivity to change within the proposed intervention context. This finding is clinically relevant, as catastrophizing is considered one of the main cognitive factors involved in the perpetuation of CMP [55]. Although the observed reduction was notable, considerable variability has been reported in studies implementing PNE alone [56,57], where improvements tend to be more limited. In contrast, programs combining PNE with TE [18,25,47] appear to yield enhanced outcomes, as observed in the present study, even with follow-up periods shorter than 6 months [53]. These findings suggest that the magnitude of change in catastrophizing may depend on factors such as intervention intensity, duration, and the integration of educational components with exercise [21,50].

Beyond the magnitude of the observed effects, the present findings warrant consideration of the potential mechanisms underlying the observed improvements, particularly in pain catastrophizing. The combination of PNE and TE may have generated a synergistic effect by simultaneously targeting cognitive and behavioral dimensions of chronic pain. PNE may have contributed to reframing maladaptive beliefs about pain, reducing perceived threat and uncertainty, while supervised therapeutic exercise provided experiential confirmation that movement could be performed safely, reinforcing these cognitive changes through embodied experience.

This synergy may be especially relevant for pain catastrophizing, a construct strongly influenced by threat appraisal, fear, and perceived lack of control. The group-based format and the progressive exposure to movement may have further enhanced these effects by normalizing pain experiences and reducing fear-avoidance behaviors.

From an implementation science perspective, delivery within the primary care setting may have facilitated these mechanisms by providing an accessible, familiar, and trusted context for patients. Integration within routine primary care physiotherapy services likely enhanced acceptability, adherence, and engagement, which are key determinants of intervention effectiveness and sustainability in real-world settings. These contextual factors may partially explain the favorable outcomes observed and support the scalability of multimodal programs combining education and exercise in primary care.

With respect to pain presence and intensity, the analyses showed statistically significant improvements in VAS scores and across all evaluated body locations, except for the right upper extremity (RUE). These findings are consistent with previous evidence indicating that multimodal physiotherapy programs produce clinically relevant reductions in pain intensity, particularly when an active approach is adopted [42,43]. In addition, the specific analysis of cervical pain revealed a corrected OR higher than that observed for other locations, which may suggest a greater probability of pain remission in this region. This finding aligns with studies reporting a favorable response of cervical pain to this type of intervention, possibly due to the influence of cognitive factors and fear of movement in this anatomical region [25,50]. Nevertheless, this interpretation should be made with caution due to the small sample size and the low frequency of events in some subgroups, which may have led to an overestimation of the effect magnitude.

The study also evaluated whether participants reported a reduction in weekly analgesic medication use. The findings suggest a sustained decrease in the use of pain-related medication. Similar to other multimodal programs with comparable characteristics [20,42,46], reliance on pharmacological treatments tends to decrease when effective coping strategies are promoted that help reduce pain and improve function, as may have occurred in the present study.

Overall, the results obtained suggest that the proposed multimodal program may have been useful in addressing both the physical mechanisms and the cognitive and perceptual processes involved in the pain experience, thereby promoting synergy between the physical and mental dimensions of quality of life discussed above. This concept is consistent with the biopsychosocial approach to chronic pain [17,46,58], in which the reduction in maladaptive cognitive and emotional factors, such as fear of movement and pain catastrophizing, may facilitate greater engagement in physical activity and contribute to broader improvements in functionality and quality of life among individuals with CMP. These changes, observed in the Pre–6 mo comparison, suggest a clinically relevant impact of the proposed multimodal approach. From this point onward, it is pertinent to examine whether the benefits achieved are maintained over the medium term.

### 4.2. Maintenance of Effects After the Program

In general, the benefits achieved following the program were maintained throughout the follow-up period, as mean differences between the Post and 6-month assessments were small and were accompanied by low effect sizes for most of the analyzed variables. This pattern suggests a relative stability of the changes achieved after program completion; however, this maintenance was not uniform across all outcomes.

Specifically, certain variables—most notably kinesiophobia (TSK-11)—showed a statistically significant deterioration between the immediate post-intervention assessment and the 6-month follow-up, although values remained improved compared with baseline. This partial erosion of benefits represents a clinically relevant finding, indicating that some domains may be more vulnerable to decline over time in the absence of ongoing support.

Some quality-of-life dimensions showed a tendency toward partial regression; however, in most cases, values remained above baseline levels. This pattern is consistent with previous literature, which suggests that educational interventions often produce rapid changes in perceptions and behaviors, whereas long-term maintenance depends on adherence to learned strategies and ongoing support [59]. This slight regression may be explained by the fluctuating nature of CMP and by the absence of a structured maintenance plan following program completion, which may limit the consolidation of benefits. Several studies have highlighted this issue [25,49]. From this perspective, the observed decline in kinesiophobia supports the inclusion of periodic reinforcement sessions in future protocols to sustain behavioral and psychosocial improvements over time. Future studies with larger samples and extended follow-up periods should explore the role of booster sessions in enhancing the durability of treatment effects.

Certain variables, such as perceived pain (VAS), analgesic consumption, kinesiophobia, and central sensitization, did not show statistically significant changes at the 6-month follow-up. This pattern may be related to the limited sample size, suggesting that future studies with larger samples are warranted to further evaluate these outcomes.

Taken together, the observed maintenance of effects appears to support the program’s capacity to produce clinically relevant changes that remain relatively stable over time, at least within the analyzed sample. Despite the stabilization of benefits, these findings further suggest the potential utility of multimodal programs based on PNE and TE as a strategy with promising effects in the management of CMP.

### 4.3. Strengths and Limitations

Given the pilot nature of this study, one of its main limitations is the sample size, which may have affected the stability of the estimates and the generalizability of the findings. In addition, the sample was highly skewed toward female participants, which limits the extrapolation of the results to male populations with chronic musculoskeletal pain. Although this sex distribution reflects the characteristics of patients commonly attending primary care physiotherapy services, it should be considered when interpreting the results.

The study also experienced an attrition rate of 16%. While adherence among participants who completed the intervention was high, losses to follow-up may have introduced attrition bias. Given the exploratory nature of this pilot study, no formal comparative analysis between participants who completed the program and those who dropped out was conducted; therefore, potential baseline differences between these groups cannot be ruled out.

Additional limitations include the absence of a control group, which prevents the establishment of firm causal relationships between the intervention and the observed changes. Moreover, the predominant use of self-reported outcome measures may have introduced recall or social desirability biases, and concomitant treatments during the follow-up period were not strictly controlled. Finally, the follow-up period was limited to 6 months; therefore, future studies should consider longer follow-up durations to confirm the long-term stability of the observed effects.

In addition, the program was delivered exclusively using the resources available within primary care physiotherapy units, without the need for external personnel or specialized equipment. This confers favorable potential in terms of cost-effectiveness and facilitates its integration into the service portfolio of these centers. Finally, implementation within the primary care setting aligns with the usual care context for patients with chronic musculoskeletal pain, promoting accessibility, continuity of care, and therapeutic adherence—key factors for optimizing clinical outcomes and maintaining effects over the medium term [23].

### 4.4. Future Directions of Research and Application to Clinical Practice

The development of this pilot study has allowed the identification of several study limitations and intervention-related weaknesses that are intended to be addressed in future research. On the one hand, conducting a study with a larger sample size and a more robust design is considered necessary in order to draw stronger and causal conclusions. Extending participant follow-up to 12 months could also provide valuable information regarding the long-term maintenance of the changes achieved through the program.

In addition, the incorporation of booster sessions or referral of participants to maintenance activities may be advisable to consolidate the clinical improvements attained and enhance their sustainability over time. Furthermore, strengthening the program through collaboration with other healthcare professionals, such as psychologists and nurses, would allow for a more comprehensive approach to the different components of CMP, potentially increasing the overall utility of the intervention. In this regard, our research team is currently working on the design of a new multidisciplinary program, the effectiveness of which will be evaluated through a randomized controlled trial.

## 5. Conclusions

A multimodal physiotherapy program incorporating pain neuroscience education and therapeutic exercise may represent a promising therapeutic approach for the management of patients with chronic musculoskeletal pain treated in primary care settings.

The findings of this pilot study suggest potential improvements in quality of life, pain catastrophizing, kinesiophobia, central sensitization, and pain intensity, consistent with a biopsychosocial perspective of pain. However, these results should be interpreted with caution, as the study was exploratory in nature and not designed to establish causal relationships or comparative efficacy.

Future studies using randomized controlled designs and longer follow-up periods are warranted to confirm these preliminary findings and to evaluate the persistence of effects over time. Additionally, the development and evaluation of maintenance strategies following program completion may be beneficial to sustain clinical gains and to promote active and self-efficacious patient engagement. Such research may help to better define the role of physiotherapy as a first-line intervention in the management of chronic pain within primary care.

## Figures and Tables

**Table 1:** Psychometric properties of the instruments used.

Instrument	Characteristics	Psychometric Properties
Health-Related Quality of Life (SF-36) [28]	36 items grouped into eight dimensions: physical functioning, role physical, bodily pain, general health, vitality, social functioning, role emotional, and mental health. Scores range from 0 to 100, with higher scores indicating better overall quality of life.	Cronbach’s alpha > 0.70. Good test–retest reliability. High correlations (r = 0.70–0.90). Good sensitivity to discriminate between different health states.
Pain Catastrophizing (Spanish version of the Pain Catastrophizing Scale, PCS) [29]	13 items grouped into three subscales: rumination, magnification, and helplessness. Total scores range from 0 to 52, with higher scores indicating greater levels of pain catastrophizing. The scale is widely used in both clinical and research settings to assess the psychological impact of pain and to guide therapeutic interventions.	Cronbach’s alpha > 0.80. Good test–retest reliability. Demonstrates significant correlations with measures of pain, disability, and mental health, supporting its convergent and predictive validity.
Kinesiophobia (Spanish version of the Tampa Scale of Kinesiophobia, TSK-11) [30]	Comprises 17 items grouped into two main dimensions: fear–avoidance and patterns of somatic hypervigilance. Total scores range from 11 to 44, with higher scores indicating greater levels of kinesiophobia.	Cronbach’s alpha ranging from 0.76 to 0.91. Test–retest reliability with coefficients above 0.80 over intervals of several weeks. Demonstrates convergent validity through significant correlations with measures of pain, functional disability, anxiety, and depression. Discriminant validity allows differentiation between individuals with different levels of fear of movement.
Central Sensitization (Spanish version of the Central Sensitization Inventory, CSI) [31]	Consists of 25 items, with total scores ranging from 0 to 100. Higher scores are associated with greater pain intensity, anxiety, depressive symptoms, somatization symptoms, disability, and sleep disturbances.	Cronbach’s alpha > 0.85. Robust test–retest reliability, with coefficients above 0.80.
Pain Intensity (Visual Analogue Scale, VAS) [32]	Measures pain intensity on a scale from 0 (no pain) to 100 (worst pain imaginable). Participants mark a point on a horizontal line that best represents the intensity of pain experienced at the time of assessment. Higher scores indicate greater pain intensity.	Demonstrates adequate construct, convergent, and discriminant validity. Additionally, it shows high sensitivity to change
Pain Location (McGill Pain Diagram) [33]	The McGill Pain Diagram, derived from the McGill Pain Questionnaire (MPQ), is a tool that allows the description and quantification of the pain experience beyond intensity alone. Pain drawings have been widely used in both clinical and research settings to record the anatomical regions in which patients report pain.	Demonstrates adequate psychometric properties in terms of reliability, validity, and sensitivity to change.
Client Satisfaction Questionnaire (CSQ-8) [34]	Assesses satisfaction with services, including healthcare services, using eight items. Total scores range from 8 to 32, with higher scores indicating greater satisfaction.	Cronbach’s alpha > 0.90 and convergent validity with perceived outcomes and treatment adherence.

**Table 2:** Descriptive analysis of sample characteristics (n = 37).

	M(SD) ^1^
Age (years)	56.19 (6.83)
Weight (kg)	74.88 (12.07)
Height (m)	1.61 (0.05)
BMI	28.89 (4.47)
	**%(n) ^2^**
**Sex**	
Male	5.4 (2)
Female	94.6 (35)
**Marital status**	
Single	5.4 (2)
Married or cohabiting union	81.1 (30)
Other unions	2.7 (1)
Separated or divorced	10.8 (4)
**Educational level**	
Primary education	48.6 (18)
Secondary education	45.9 (17)
Higher education	5.4 (2)
**Employment status**	
Employed	43.2 (16)
Retired	24.3 (9)
Unemployed	32.4 (12)

^1^: mean (standard derivation); ^2^: relative frequencies (absolute frequencies).

**Table 3:** Intragroup comparison results.

	Pre–6 mo						Post–6 mo		
	Pre %(n)	Post %(n)	6 mo %(n)	Mean Difference (95% CI) *	Effect Size (Cohen’s d)	MCID %(n)	Mean Difference (95% CI) *	Effect Size (Cohen’s d)	MCID %(n)
**SF-36**									
Physical Functioning	50.13 (19.87)	66.35 (22.10)	62.02 (22.95)	−11.89 (−17.65–−6.12) *	−0.68 (−1.04–−0.32)	78.4 (29)	2.90 (−1.57–10.22)	0.24 (−0.08–0.57)	35.1 (13)
Role Limitation Physical	31.97 (20.28)	55.75 (26.91)	51.83 (26.10)	−19.86 (−27.22–−12.50) *	−0.90 (−1.27–−0.51)	78.4 (29)	3.91 (−3.16–10.99)	0.18 (−0.14–0.50)	35.1 (13)
Role Limitation Emotional	55.86 (26.56)	69.81 (30.54)	66.67 (29.47)	4.75 (−20.44–−1.17) *	−0.37 (−0.70–−0.03)	45.9 (17)	3.13 (−3.84–10.11)	0.15 (−0.17–0.47)	24.3 (9)
Vitality	24.86 (19.94)	52.29 (25.07)	44.18 (24.42)	−19.32 (−25.90–−12.74) *	−0.98 (−1.36–−0.58)	78.4 (29)	8.10 (1.43–14.78) *	0.40 (0.06–0.73)	27.0 (10)
Role Emotion	50.05 (22.10)	71.13 (20.29)	61.45 (22.09)	−11.35 (−19.48–−3.21) *	−0.46 (−0.80–−0.12)	59.5 (22)	9.73 (4.70–14.75) *	0.64 (0.28–0.99)	10.8 (4)
Social Functioning	45.13 (23.29)	68.37 (27.23)	63.73 (28.54)	−18.59 (−28.18–−9.09) *	−0.64 (−0.99–−0.28)	67.6 (25)	4.64 (−1.17–10.47)	0.26 (−0.06–0.59)	24.3 (9)
Body Pain	27.45 (15.60)	48.08 (24.04)	41.32 (23.22)	−13.86 (−20.26–−7.46) *	−0.72 (−1.08–−0.35)	54.1 (20)	6.75 (−0.84–14.35)	0.29 (−0.03–0.62)	24.3 (9)
General Health	39.59 (16.43)	55.27 (18.44)	49.37 (20.44)	−10.13 (−13.89–−6.37) *	−0.89 (−1.27–-0.51)	78.4 (29)	5.54 (1.29–9.78) *	0.43 (0.09–0.77)	29.7 (11)
**TSK-11**	28.16 (7.27)	19.35 (6.82)	23.05 (6.76)	5.10 (2.58–7.63) *	0.67 (0.31–1.02)	51.4 (19)	−3.70 (−5.25–−2.15) *	−0.79 (−1.16–−0.42)	2.7 (1)
**CSI**	52.43 (13.90)	40.75 (14.94)	43.27 (16.21)	9.16 (4.34–13.97) *	0.63 (0.27–0.98)	64.9 (24)	−2.5 (−5.61–0.59)	−0.27 (−0.59–0.06)	13.5 (5)
**PCS**	34.70 (8.62)	23.32 (11.63)	24.40 (10.64)	10.29 (7.60–12.98) *	1.27 (0.83–1.70)	64.9 (24)	−1.08 (−3.70–1.54)	−0.13 (−0.46–0.18)	21.6 (8)
Rumination PCS	12.05 (2.65)	8.37 (3.70)	8.86 (3.91)	3.18 (2.16–4.21) *	1.04 (0.63–1.43)	73.0 (27)	−0.49 (−1.47–0.50)	−0.16 (−0.48–0.16)	29.7 (11)
Magnification PCS	6.91 (2.30)	4.59 (2.59)	4.75 (2.88)	2.16 (1.30–3.01) *	0.84 (0.46–1.21)	59.5 (22)	−0.16 (−0.92–0.59)	−0.07 (−0.39–0.25)	16.2 (6)
Helplessness PCS	15.73 (4.74)	10.35 (6.33)	10.78 (5.06)	4.94 (3.51–6.38) *	1.15 (0.72–1.56)	75.7 (28)	−0.43 (−2.06–1.20)	−0.08 (−0.41–0.23)	32.4 (12)
**VAS**	57.67 (14.98)	41.56 (17.62)	41.54 (21.86)	16.13 (9.77–22.49) *	0.84 (0.46–1.21)	56.8 (21)	0.02 (−5.08–5.14)	0.01 (−0.32–0.32)	16.2 (6)
**Weekly analgesic consume**	22.33 (17.84)	13.01 (13.41)	13.37 (19.58)	8.95 (3.80–14.11) *	0.57 (0.22–0.92)	73 (27)	−0.36 (−3.90–3.17)	−0.03 (−0.35–0.28)	32.4 (12)
**Healthcare utilization**	-	0.67 (0.88)	1.10 (1.48)	-	-	-	−0.43 (−0.96–0.10)	−0.26 (−0.59–0.06)	16.2 (6)
**CSQ-8**	-	29.29 (2.64)	29.32 (2.57)	-	-	-	−0.02 (−0.78–0.72)	−0.01 (−0.33–0.31)	13.5 (5)

Pre: baseline values; Post: post-intervention values (week 12); 6 mo: values at 6 months after program completion; MCID: minimal clinically important difference; * statistically significant intragroup differences (*p* < 0.05).

**Table 4:** Results of pain location analysis.

	Pre	Post	6 Months	Pre–6 Months			Post–6 Months		
	%(n)	%(n)	%(n)	*p*	Δ%	OR (IC95%)	*p*	Δ%	OR (IC95%)
**C-spine**									
Yes	89.2 (33)	75.7 (28)	63.9 (23)	0.002	−25.3	19 (1.10–326.5) +	0.248	−11.8	2 (0.60–6.64)
No	10.8 (4)	24.3 (9)	36.1 (13)						
**L-spine**									
Yes	86.5 (32)	70.3 (26)	63.9 (23)	0.011	−22.6	9 (1.14–71.04)	0.563	−6.4	1.4 (0.44–0.41)
No	13.5 (5)	29.7 (11)	36.1 (13)						
**T-spine**									
Yes	51.4 (19)	32.4 (12)	27.0 (10)	0.020	−24.4	4 (1.12–14.17)	0.593	8.1	1.3 (0.46–3.86)
No	48.6 (18)	67.6 (25)	27 (73.0)						
**RUE**									
Yes	56.8 (21)	48.6 (18)	50.0 (18)	0.414	−6.8	2 (0.36–0.92)	1.000	1.4	1 (0.20–4.95)
No	43.2 (16)	51.4 (19)	50.0 (18)						
**LUE**									
Yes	67.6 (25)	35.1 (13)	43.2 (16)	0.020	−24.4	4 (1.12–14.17)	0.317	8.1	0.5 (0.12–1.99)
No	32.4 (12)	64.9 (24)	56.8 (21)						
**RLE**									
Yes	73.0 (27)	56.8 (21)	50.0 (18)	0.020	−23	5 (1.09–22.82)	0.317	−6.8	2 (0.50–7.99)
No	27.0 (10)	43.2 (16)	50.0 (18)						
**LLE**									
Yes	73.0 (27)	59.5 (22)	50.0 (18)	0.011	−23	9 (1.14–71.04)	0.248	−9.5	2 (0.60–6.64)
No	27.0 (10)	40.5 (15)	50.0 (18)						

Pre: baseline values; Post: post-intervention values (week 18); 6 months: values at 6 months after program completion; %(n): frequencies; *p*: *p*-value (McNemar test); Δ: difference in proportions; OR (95% CI): odds ratio (95% confidence interval); +: corrected OR.

## Data Availability

Dataset available on request from the authors. The data are not publicly available due to privacy and ethical restrictions.

## Supplementary Materials

The following supporting information can be downloaded at https://www.mdpi.com/article/10.3390/healthcare14040456/s1.

## Abbreviations

BMI	Body Mass Index
CMP	Chronic Musculoskeletal Pain
CSI	Central Sensitization Inventory
CSQ-8	Customer Satisfaction Questionnaire
C-spine	Cervical Spine
GDP	Gross Domestic Product
LLE	Left Lower Extremity
LUE	Left Upper Extremity
L-spine	Lumbar Spine
MCID	Minimal Clinically Important Difference
OR	Odds Ratio
PNE	Pain Neuroscience Education
RLE	Right Lower Extremity
RUE	Right Upper Extremity
SF	Short Form
TE	Therapeutic Exercise
TREND	Transparent Reporting of Evaluations with Nonrandomized Designs
TSK-11	Tampa Scale of Kinesiophobia
T-spine	Thoracic Spine
VAS	Visual Analogic Scale
YLDs	Years Lived with Disability

## Appendix A

The intervention consisted of a multimodal physiotherapy program comprising two components: PNE and TE. It was delivered in the physiotherapy units of the two participating primary care centers and spanned a total of 12 consecutive weeks (see Figure A1).

The intervention was conducted in a group format, with each group consisting of a minimum of 8 and a maximum of 12 participants, and was led by a physiotherapist with clinical experience in the management of chronic musculoskeletal pain and prior training in pain neuroscience education. All clinicians followed a standardized intervention protocol to ensure consistency across sessions and study sites. The program content was based on similar and pioneering experiences developed in Spain, which have been supported by high-impact publications [25,60].

The first component of the program consisted of PNE delivered over five group sessions of 90 min each, conducted once per week during weeks 1 to 5. Each session combined theoretical content with interactive activities aimed at facilitating understanding and active engagement. Visual aids, infographics, and short educational videos were used to reinforce key concepts and enhance motivation. All sessions were delivered through structured presentations created using PowerPoint software (v.16.105.3) and adapted to the educational level of the participants.

The core educational content focused on contemporary pain neuroscience concepts, including the distinction between acute and chronic pain, central sensitization mechanisms, the role of the nervous system in pain modulation, and the influence of cognitive, emotional, and contextual factors on pain perception. Specific core messages emphasized that pain does not necessarily reflect tissue damage, that the nervous system can become hypersensitive over time, and that pain is a modifiable experience influenced by movement, thoughts, emotions, and lifestyle behaviors. Common maladaptive beliefs related to fear of movement, catastrophizing, and passive coping strategies were explicitly addressed and reframed.

Interactive components included group discussions, reflective questions, practical examples related to daily activities, and brief experiential exercises designed to encourage participants to relate the educational content to their own pain experience. The primary objective of this phase was to modify maladaptive beliefs about pain, reduce fear-avoidance behaviors, and increase participants’ readiness to engage in active self-management strategies.

This educational phase was designed to prepare participants for the second component of the intervention (therapeutic exercise), corresponding to the “action” stage of behavior change according to the Transtheoretical Model proposed by Prochaska and DiClemente [61,62].

The second component of the program consisted exclusively of supervised group-based TE sessions, delivered over 18 sessions of 90 min each, conducted three times per week between weeks 6 and 11. All sessions followed the structure recommended by the American College of Sports Medicine [63], ensuring safety, progression, and individual adaptation.

Each session included three phases: (i) a warm-up phase (5–10 min) consisting of low-intensity aerobic activities (e.g., walking or mobility exercises) and joint mobility exercises; (ii) a main exercise phase (35–45 min) focused on multicomponent therapeutic exercise; and (iii) a cool-down phase (10–15 min) including stretching, breathing exercises, and relaxation techniques.

The main exercise phase incorporated a combination of strength training, motor control exercises, functional movements, and low-impact aerobic exercises. Strength exercises targeted major muscle groups using body weight, elastic resistance bands, and light external loads, with an emphasis on proper technique and gradual load progression. Motor control and functional exercises were designed to improve movement quality, coordination, and postural control during daily activities. Aerobic exercises were performed at low to moderate intensity, adjusted according to participants’ tolerance and perceived exertion.

Exercise intensity and complexity were progressively increased throughout the program, following the principles of gradual overload and individualization. Pain-related fear and avoidance behaviors were actively addressed by encouraging safe movement, emphasizing symptom-guided progression rather than pain avoidance, and reinforcing the educational messages delivered during the PNE component.

The primary objectives of this component were to restore functional capacity, increase confidence in movement, and promote physical activity engagement, with the subsequent goal of progressively reducing pain intensity and improving overall physical and psychosocial functioning.

To conclude the program, a booster session lasting 120 min was conducted in week 12. This session aimed to reinforce key principles of TE, review correct execution of the main exercises, and address participants’ questions. In addition, core pain PNE concepts were revisited to consolidate understanding of pain mechanisms, promote adaptive beliefs, and support the continued application of self-management strategies. This final session was designed to encourage long-term engagement in physical activity and to facilitate the maintenance of the benefits achieved during the supervised intervention period.
