# Supplementary figures and images for: “Learn and Move” Program for the Management of Chronic Musculoskeletal Pain in Primary Care: A Pilot Study
Source: Healthcare (Basel). 2026 Feb 11;14(4):456. doi: 10.3390/healthcare14040456 (PMC12941048; doi:10.3390/healthcare14040456)

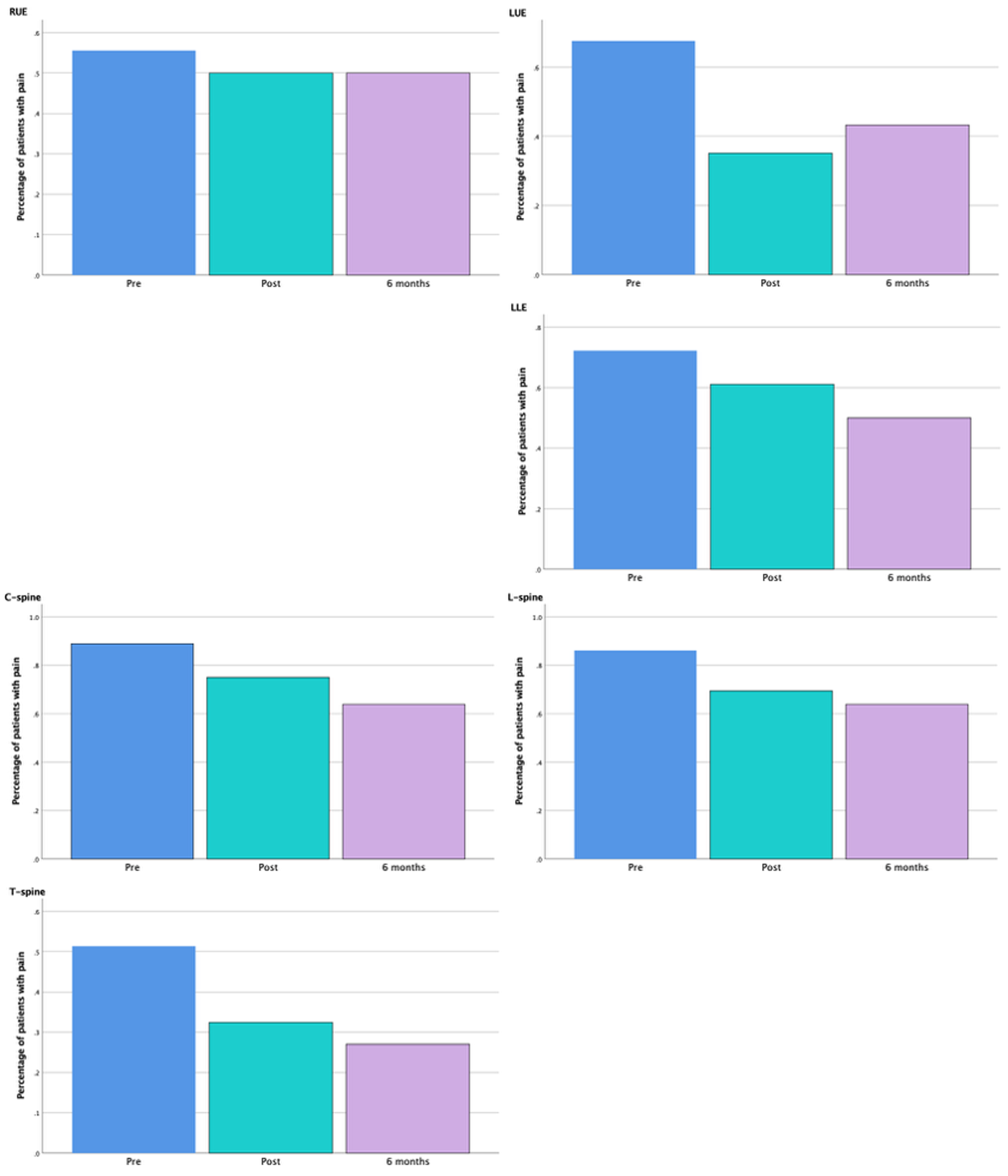

Supplement: Supplementary file 1 [file healthcare-14-00456-s001.zip › Supplementary material S1.png]
